# ROCK inhibition as a therapy for spinal muscular atrophy: understanding the repercussions on multiple cellular targets

**DOI:** 10.3389/fnins.2014.00271

**Published:** 2014-08-28

**Authors:** Emmanuelle Coque, Cédric Raoul, Mélissa Bowerman

**Affiliations:** ^1^The Institute for Neurosciences of Montpellier, Saint Eloi Hospital, Institut National de la Santé et de la Recherche Médicale UMR1051Montpellier, France; ^2^Université de Montpellier 1 and 2Montpellier, France

**Keywords:** spinal muscular atrophy, RhoA, ROCK, Y-27632, Fasudil

## Abstract

Spinal muscular atrophy (SMA) is the most common genetic disease causing infant death, due to an extended loss of motoneurons. This neuromuscular disorder results from deletions and/or mutations within the *Survival Motor Neuron 1* (*SMN1*) gene, leading to a pathological decreased expression of functional full-length SMN protein. Emerging studies suggest that the small GTPase RhoA and its major downstream effector Rho kinase (ROCK), which both play an instrumental role in cytoskeleton organization, contribute to the pathology of motoneuron diseases. Indeed, an enhanced activation of RhoA and ROCK has been reported in the spinal cord of an SMA mouse model. Moreover, the treatment of SMA mice with ROCK inhibitors leads to an increased lifespan as well as improved skeletal muscle and neuromuscular junction pathology, without preventing motoneuron degeneration. Although motoneurons are the primary target in SMA, an increasing number of reports show that other cell types inside and outside the central nervous system contribute to SMA pathogenesis. As administration of ROCK inhibitors to SMA mice was systemic, the improvement in survival and phenotype could therefore be attributed to specific effects on motoneurons and/or on other non-neuronal cell types. In the present review, we will present the various roles of the RhoA/ROCK pathway in several SMA cellular targets including neurons, myoblasts, glial cells, cardiomyocytes and pancreatic cells as well as discuss how ROCK inhibition may ameliorate their health and function. It is most likely a concerted influence of ROCK modulation on all these cell types that ultimately lead to the observed benefits of pharmacological ROCK inhibition in SMA mice.

## Introduction

Spinal muscular atrophy (SMA) is a devastating neurodegenerative disease, affecting approximately 1:6,000–10,000 live births per year (Pearn, 1978; Crawford and Pardo, 1996). This autosomal recessive disease, a major inherited cause of infant and child death (<2 years old), is characterized by the loss of spinal alpha motoneurons, muscle weakness, atrophy and subsequent paralysis (Crawford and Pardo, 1996). The clinical severity of SMA is characterized into 4 types: type 1 is the most severe as it primarily affects newborns, type 2 and type 3 are intermediate forms with a childhood onset, and type 4 is symptomatically the mildest with an adult onset (Pearn, 1980; Munsat and Davies, 1992).

SMA is due to homozygous mutations and/or deletions in the *Survival Motor Neuron 1* (*SMN1*) gene and subsequent reduction of the SMN protein (Lefebvre et al., 1995). While most species only have one copy of the gene, humans have two: a telomeric copy (*SMN1)* and a duplicated centromeric copy (*SMN2)*, both located on chromosome 5q13 (Lefebvre et al., 1995; DiDonato et al., 1997). While *SMN1* and *SMN2* differ by a few nucleotides, the C to T substitution in exon 7 of *SMN2* is critical (Lorson et al., 1999). Indeed, it leads to the loss of an exon splicing enhancer or the gain of an exon splicing silencer that leads to the production of a truncated, non-functional SMN protein that lacks exon 7, named *SMN*Δ*7* (Lefebvre et al., 1995; Cartegni and Krainer, 2002; Kashima and Manley, 2003). As a result, *SMN*Δ*7* is the major product of *SMN2* and the number of copies of *SMN2* is linked to the severity of the disease (Lefebvre et al., 1997).

Based on this genetic knowledge, a first mouse model for SMA was created with a homozygous deletion of the *Smn* gene (Schrank et al., 1997). This null mutant, *Smn*^−/−^, served to highlight SMN as a developmentally essential protein since its complete ablation is embryonic lethal (Schrank et al., 1997) The heterozygous *Smn*^+/−^ however, does not develop the typical histopathological SMA hallmarks and remains at best a hypomorphic model for the disease (Schrank et al., 1997; Bowerman et al., 2014). Thus, several alternate SMA models were eventually created in order to more closely mimic the human pathology by either introducing partially functional human SMN constructs onto the *Smn*^−/−^ background or by rendering the endogenous murine *Smn* gene similar to the *SMN2* copy (reviewed in Bebee et al., 2012). At present, the most commonly used SMA models are termed *Smn*^−/−^; *SMN2* (Hsieh-Li et al., 2000; Monani et al., 2000), *Smn*^−/−^; *SMN2*; *SMN*^Δ7/Δ7^ (Le et al., 2005) and *Smn*^2*B*/−^ mice (Hammond et al., 2010; Bowerman et al., 2012a) and range from severe (death within the first post-natal week) to intermediate phenotypes (average lifespan of 30 days). In spite of these differences in survival times, all of these models display spinal cord motoneuron degeneration, muscle atrophy as well as neuromuscular junction (NMJ) defects and have therefore been indispensable in our understanding of the disease and in the evaluation of potential therapeutic approaches.

The SMN protein is found in both the cytoplasm and the nucleus, where in the latter, it is concentrated in structures called Cajal bodies and Gemini of coiled bodies (Gems) (Liu and Dreyfuss, 1996; Carvalho et al., 1999). The best described housekeeping role for SMN is in the cytoplasmic assembly of small nuclear ribonucleoproteins (snRNPs), essential components of the pre-mRNA spliceosome (reviewed in Burghes and Beattie, 2009). Once snRNPs are correctly assembled, the SMN-snRNP complex is imported in the nucleus where it localizes to Cajal bodies for further processing and maturation (reviewed in Morris, 2008). While splicing defects have been observed in SMA mice (Zhang et al., 2008; Baumer et al., 2009; Lotti et al., 2012), none have been qualified as being solely responsible for the pathology of the disease. Hence the myriad of studies aimed at uncovering specific neuronal roles for Smn (reviewed in Boyer et al., 2010). One of these functions is the modulation of the actin cytoskeleton, revealed by the initial report that Smn was responsible for β-actin mRNA localization in motoneuron growth cones (Rossoll et al., 2003). Subsequent studies in neuronal cells and/or in spinal cord extracts have further demonstrated a direct or indirect interaction between Smn and molecular modulators of actin dynamics such as profilin, plastin 3, small Rho GTPases RhoA and Cdc42 as well as Rho kinase (ROCK), a direct downstream effector of RhoA (Giesemann et al., 1999; Bowerman et al., 2007; Oprea et al., 2008; Nolle et al., 2011). Interestingly, although the genetic manipulation of profilin and plastin 3 did not improve the lifespan of SMA mice (Bowerman et al., 2009; Ackermann et al., 2013), the pharmacological targeting of ROCK (via inhibitors Y-27632 and Fasudil), a more upstream regulator of actin dynamics, led to a significant increase in survival (Bowerman et al., 2010, 2012b). The beneficial effect of ROCK inhibition on lifespan was accompanied by an augmentation of muscle fiber size and an increased morphological maturation of the NMJ, without impacting the number of surviving motoneurons or the expression level of Smn. While the spinal cord, and more specifically the motoneurons, were the primary therapeutic targets of ROCK inhibition in SMA mice, the systemic delivery approaches used in these studies undeniably result in non-neuronal modulation of the ubiquitously expressed RhoA/ROCK pathway. Further, although motoneurons are the most vulnerable cell type in SMA, an increasing number of reports suggest that other cells and tissues are also affected by loss of SMN (reviewed in Hamilton and Gillingwater, 2013).

**Figure 1:**
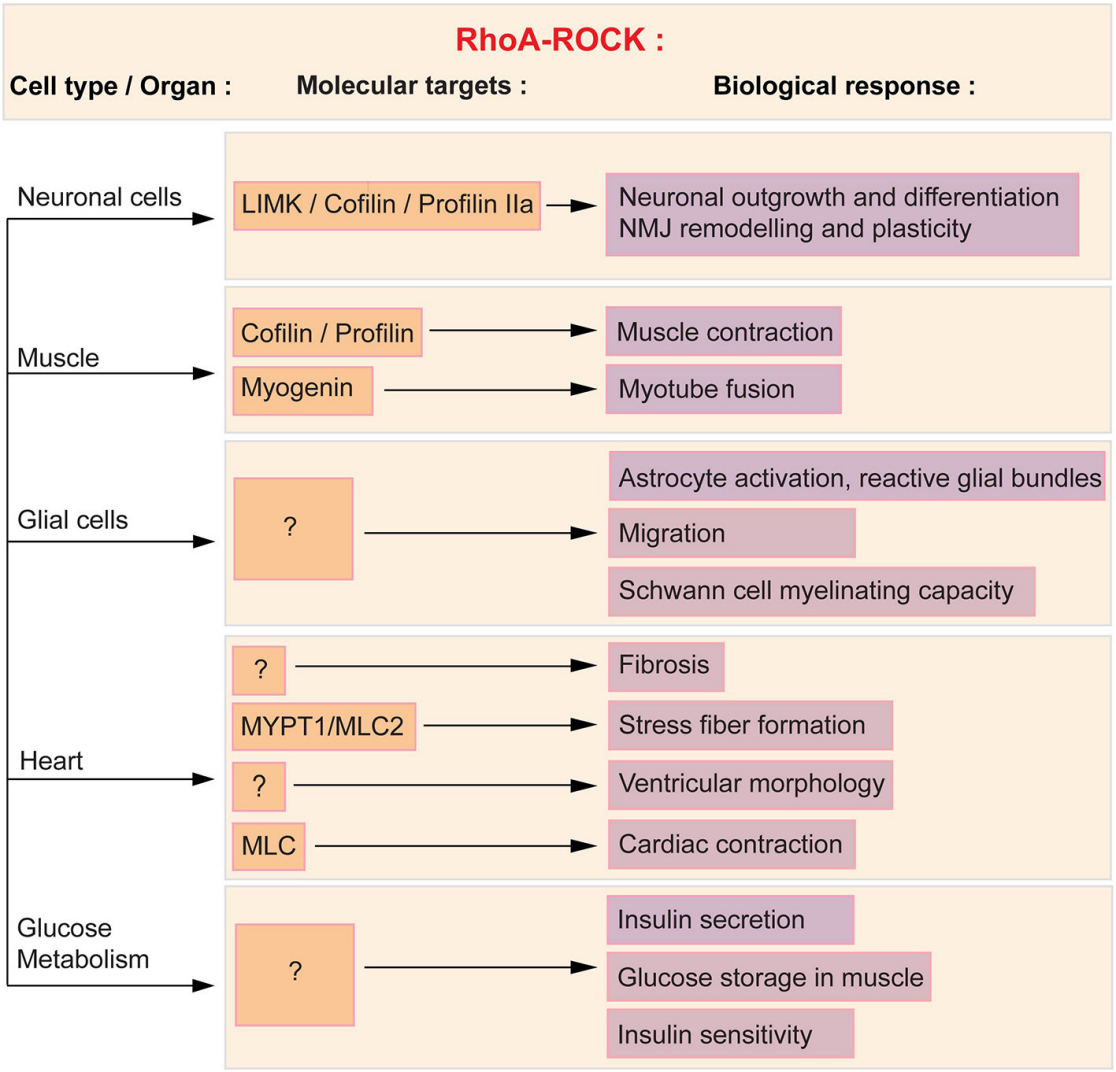
**RhoA/ROCK molecular and functional targets in affected SMA cells and tissues**. ROCK acts in a variety of cells and tissues via several downstream effectors, of which some are known and described herein (orange boxes) and subsequently linked to the associated cellular process (purple boxes). Cofilin, profilin and LIMK regulate neuronal outgrowth and differentiation as well as NMJ plasticity. Cofilin, profilin and myogenin mediate the effect of RhoA in muscle. In glial cells, the downstream effectors of the RhoA/ROCK pathway have yet to be defined. Cardiac physiology and function is in part influenced by myosin phosphatase (MYPT1) and myosin light chain 2 (MLC2). Both proteins regulate stress fiber formation and cardiac contraction. Finally, the molecular intermediates in the regulation of glucose and fatty acid metabolism have yet to be elucidated.

**Figure 2:**
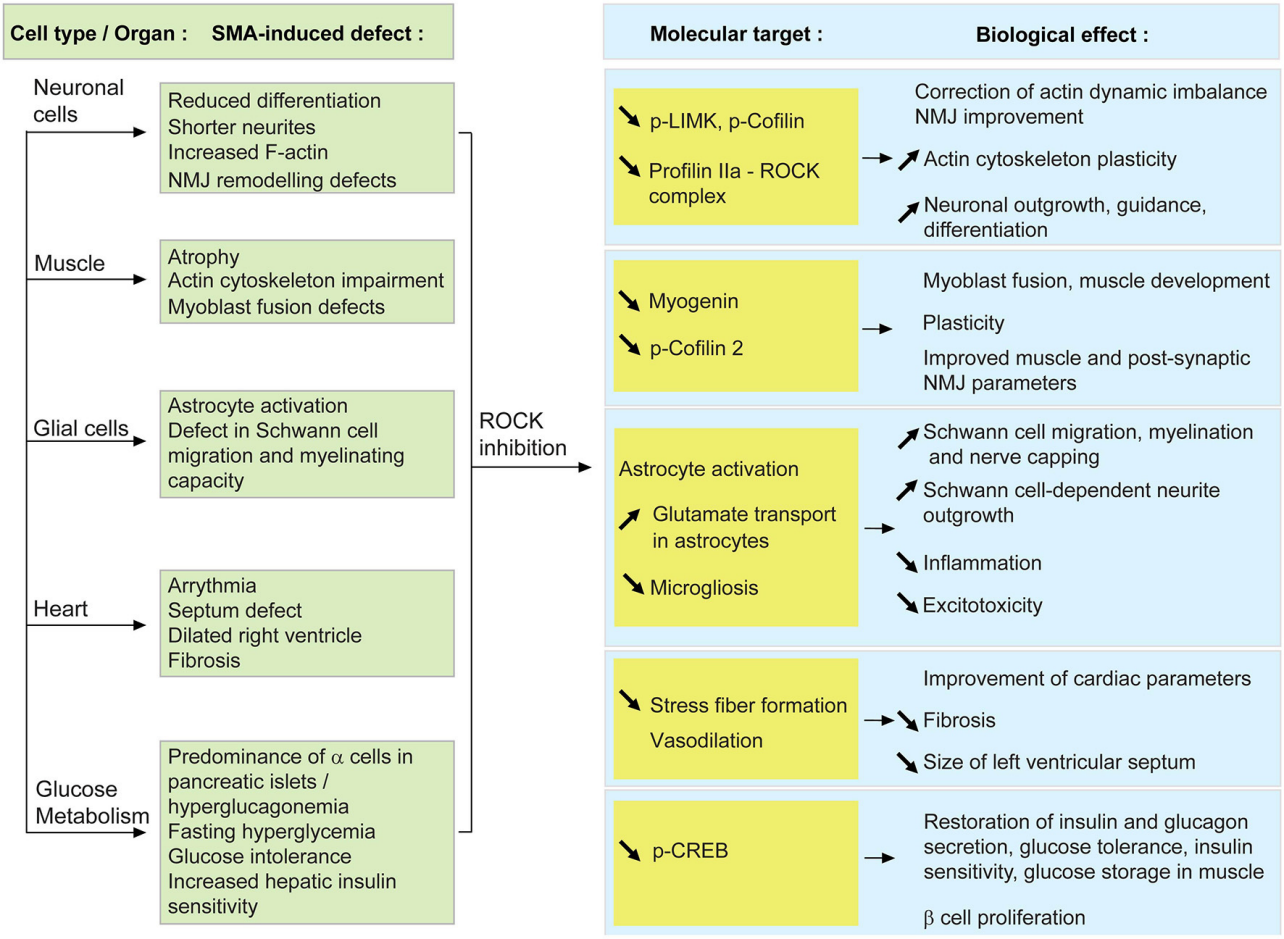
**Proposed model of how systemic ROCK inhibition in SMA mice may result in beneficial effects within several cells and tissues**. When SMN is depleted, the activity of the RhoA/ROCK pathway is increased which leads to several cellular defects (described in the green boxes). Upon systemic ROCK inhibition, these aberrant pathways are ameliorated in neurons, muscle, glial cells, heart and pancreas (blue boxes). The known or putative molecular intermediates responsible for mediating the improvements are cited in the yellow boxes.

In the present review, we will discuss the potential cellular targets that may participate in the beneficial effect of ROCK inhibition in SMA mice, To this aim, the role of the RhoA/ROCK pathway in healthy cells and in SMA pathology will be analyzed (Figure 1) and we will elaborate on how the systemic modulation of ROCK could influence these cells and tissues, and potentially the course of the disease (Figure 2).

## Neuronal cells

### RhoA/ROCK, neuronal cells and SMA

The primary cellular target in SMA is undeniably the spinal cord motoneuron. In neuronal cells, the modulation of the actin cytoskeleton by the RhoA/ROCK pathway affects growth, neurite formation, polarization, branching, regeneration, pathfinding, guidance and retraction (reviewed in Luo et al., 1997; Govek et al., 2005). Whether neuronal or non-neuronal, the RhoA/ROCK signaling cascade implicates various downstream effectors such as cofilin, myosin light chain phosphatase (MYPT), profilin IIa, Lim kinases (LIMK) and myosin regulatory light chain (MLC) (reviewed in Schofield and Bernard, 2013).

The first evidence linking SMN and the RhoA/ROCK pathway was reported in the neuronal-like PC12 cells (Bowerman et al., 2007). Knocking down *Smn* in PC12 cells by RNA interference (RNAi) lead to a reduced differentiation potential, shorter neurites and more importantly, perturbation of the actin cytoskeleton and key modulators of actin dynamics. Indeed, in Smn-depleted PC12 cells, there is an increase in RhoA-GTP, the active form of the kinase, which is known to inhibit neuronal outgrowth and differentiation (reviewed in Hall, 1998; Mueller, 1999). Downstream effectors of the RhoA/ROCK pathway such as cofilin, MYPT and profilin IIa (Kimura et al., 1996; Sumi et al., 1999; Da Silva et al., 2003) are also aberrantly expressed and/or phosphorylated in Smn-depleted PC12 and NCS34 neuronal-like cells (Bowerman et al., 2007; Nolle et al., 2011; Hensel et al., 2014). Of interest is the interaction of both ROCK and SMN with profilin IIa (Giesemann et al., 1999; Da Silva et al., 2003; Sharma et al., 2005; Nolle et al., 2011), the neuronal isoform of the *profilin II* gene, which plays a key role in the control of actin stability (Di Nardo et al., 2000; Lambrechts et al., 2000). Importantly, the expression and phosphorylation of profilin IIa is upregulated in Smn-depleted PC12 cells and at the same time, the amount of ROCK-profilin IIa complex is increased (Bowerman et al., 2007; Nolle et al., 2011). The interaction between RhoA, ROCK and phosphorylated profilin IIa has previously been shown to play an inhibitory role on actin-mediated neuronal outgrowth and differentiation (Da Silva et al., 2003). A similar augmentation of RhoA-GTP was observed in the spinal cord of an intermediate SMA mouse model at both pre-symptomatic and symptomatic stages (Bowerman et al., 2010). Taken together, these results highlight an increased activity of the RhoA/ROCK pathway in Smn-depleted neuronal cells and tissue. Seeing as RhoA/ROCK are key regulators of actin dynamics (reviewed in Hall, 1998), the ensuing aberrantly regulated actin cytoskeleton may hinder neuronal outgrowth, differentiation and/or plasticity of SMN-depleted neurons (Figure 1).

### Potential benefits of ROCK inhibition on neuronal cells

When treated with a ROCK inhibitor (Y-27632 or Fasudil), the lifespan of an intermediate SMA mouse model is dramatically increased (Bowerman et al., 2010, 2012b). Since levels of Smn protein and mRNA are unchanged by these pharmacological compounds, we can presume that the observed effects are directly due to the inhibition of the ROCK pathway itself and not to an indirect impact on the expression of Smn. Moreover, the treatment of SMA mice with Y-27632 and Fasudil did not induce an increase in the number of surviving motoneurons, suggesting that the beneficial effects of ROCK inhibitors are most likely on improving the health and function of the remaining motoneurons, but not on preventing the SMN-dependent NMJ denervation and subsequent axonal and neuronal loss.

At a molecular level, there is a reduction of p-LIMK and p-cofilin, downstream effectors of ROCK (Maekawa et al., 1999; Sumi et al., 1999), in SMA mice treated with ROCK inhibitors (Bowerman et al., 2010, 2012b). Decreased levels of p-LIMK and p-cofilin could hence create a favorable environment to correct the existing actin polymerization imbalance. Despite the fact that profilin IIa levels were not assessed in the spinal cord of SMA mice treated with ROCK inhibitors, we can hypothesize a similar effect on profilin IIa to the ones reported for LIMK and cofilin. Since ROCK is known to form a complex with profilin IIa (Da Silva et al., 2003), we can also propose that inhibition of ROCK decreases levels of profilin IIa-ROCK complex, thus allowing for a more plastic actin cytoskeleton (Figure 2).

While inactivation of the ROCK pathway promotes neuronal outgrowth and differentiation as well as guidance (reviewed in Hall, 1998; Mueller, 1999), previous studies have shown that in SMA mice, motoneurons adequately reach and innervate their muscle target (Murray et al., 2008; Ling et al., 2012). A recent report however, suggests that Smn-depleted axons have a reduced remodeling capacity following injury (Murray et al., 2013). Thus, ROCK inhibition in SMA mice may increase actin plasticity in nerve terminals, therefore improving their adaptive ability during the neurodegenerative process and therefore increasing the maintenance of functional NMJs (Figure 2). Moreover, as mentioned above, treatment with ROCK inhibitors decreases expression of p-LIMK in spinal cord of SMA mice (Bowerman et al., 2010). This kinase is known to play a role at the NMJ, suppressing synaptic sprouting and growth (Ang et al., 2006). The downregulation of p-LIMK following inhibition of the ROCK pathway could therefore improve the plasticity of pre-synaptic NMJ terminals. However, this does not appear to be the case as analysis of NMJs from Fasudil-treated SMA mice reveals that ROCK inhibition does not improve pre-synaptic pathology such as neurofilament accumulation and denervation (Bowerman et al., 2012b). Then again, the effect of decreased p-LIMK on synaptic sprouting and growth may be slow yet still beneficial over time, eventually leading to the occurrence of completely normal NMJs in aging Fasudil-treated SMA mice (Bowerman et al., 2012b).

## Skeletal muscle

### RhoA/ROCK, muscle physiology and SMA

Muscle weakness and atrophy are also principal pathological SMA hallmarks. Several studies using C2C12 myoblast-like cells (Shafey et al., 2005), conditional knockout mouse models (Cifuentes-Diaz et al., 2001; Nicole et al., 2003) as well as human and murine primary myoblast cultures (Arnold et al., 2004; Boyer et al., 2014) have highlighted an intrinsic role for Smn in myoblast fusion and proliferation as well as in the correct formation of myotubes.

In muscle, the RhoA/ROCK pathway is required for proper muscle contraction and function (Formigli et al., 2004) as well as skeletal myogenesis (reviewed in Bryan et al., 2005). While to date no study has assessed the expression of RhoA and ROCK in SMA muscle, the impact of Smn depletion on the expression of downstream effectors of ROCK has been assessed. Indeed, the expression of the muscle-specific p-cofilin 2 (Ono et al., 1994) is increased in the *tibialis anterior* (TA) of SMA mice (Bowerman et al., 2012b), suggesting that the RhoA/ROCK pathway could also be misregulated in this tissue. Cofilin is an actin-binding protein, which disassembles actin filaments when in its active non-phosphorylated form (reviewed in Mizuno, 2013). Upregulation of active cofilin could thus affect actin polymerization, promoting the more stable filamentous (F) form of actin, and subsequently affecting muscle physiology and function (Figure 1). However, the hypothesis for a ROCK-dependent misregulation of p-cofilin 2 in SMA muscle does require further exploration since other proteins can activate the LIMK-cofilin 2 pathway (reviewed in Maciver and Hussey, 2002).

### Potential benefits of ROCK inhibition on skeletal muscle

When ROCK inhibitors were administered to an intermediate SMA mouse model, several prominent modifications at the muscular level were observed (Bowerman et al., 2010, 2012b). Indeed, administration of Fasudil and Y-27632 lead to increased TA myofiber size and NMJ endplate area as well as improved morphological maturity, without any influence on motoneuron number. These results suggest that the positive effects of ROCK inhibitors may not be mediated by motoneurons but by the muscle itself. It is interesting to note that this gain in muscle myofiber size was not correlated with an increase in overall weight. However, such a phenomenon has already been described in other cases of muscle atrophy or hypertrophy (Rehfeldt et al., 2005; Knapp et al., 2006; Kraemer and Vladimir, 2006). Despite this absence of weight gain, mice treated with ROCK inhibitors still display a significant improvement in survival, suggesting that modulation of intrinsic muscle parameters such as expression of myogenic regulatory factors and actin-dependent regulation of myogenesis, is sufficient to ameliorate disease progression in SMA mice (Figure 2).

Cofilin is not the only RhoA/ROCK-dependent misregulated protein in SMA mouse muscle. Myogenin is a muscle-specific transcription factor, member of the MyoD family and required for fusion of myogenic precursor cells during myogenesis (Hasty et al., 1993). Interestingly, myogenin is upregulated in skeletal muscle of SMA mice as well as in Smn-depleted primary myoblasts (Bowerman et al., 2012b; Boyer et al., 2014; Bricceno et al., 2014). As active RhoA has been shown to positively control the expression of myogenin (Takano et al., 1998; Dhawan and Helfman, 2004), we could hypothesize that the increased expression of myogenin is a consequence of the upregulated RhoA/ROCK pathway in SMA muscle. This could explain the myoblast fusion defects observed in patient- and mouse-derived Smn-depleted myoblasts and Smn C2C12 cells (Arnold et al., 2004; Shafey et al., 2005; Boyer et al., 2014). At a molecular level, by returning the expression of myogenin to physiological levels, ROCK inhibition could promote normal muscle development, thus explaining the improvement in myofiber size.

As discussed earlier, the administration of ROCK inhibitors to SMA mice dramatically increases myofiber and NMJ endplate area (Bowerman et al., 2010, 2012b). It has therefore been suggested that this amelioration in post-synaptic morphology could have a positive effect on the ensuing synaptic connection (reviewed in Sanes and Lichtman, 1999), hence protecting the surviving motoneurons. Further, the modulation of actin dynamics by ROCK inhibition could have an effect on acetylcholine receptor clustering, which is a crucial actin-dependent step for correct NMJ formation (Dobbins et al., 2006; Cartaud et al., 2011). Taken together, these observations raise the possibility that while improving skeletal muscle and NMJ parameters, ROCK inhibition could also positively affect the function of the remaining motoneurons. Conversely, the overall benefit of ROCK inhibition in SMA mice may result from the combinatory effects in neuron and muscle, resulting in an improved communication and maintenance via the NMJ.

## Glial cells

### RhoA/ROCK, glial cells and SMA

Glial cells such as astrocytes, microglia and Schwann cells are essential to maintain functional homeostasis within the nervous system as they provide physical support for neurons and secrete trophic factors important for neuronal health (reviewed in Fields and Stevens-Graham, 2002; Allen and Barres, 2009). Conversely, they can also contribute to the neurodegenerative process when they become aberrantly activated (reviewed in Ilieva et al., 2009).

While limited, previous studies have identified glial cell abnormalities in SMA patients and mouse models as well as in Smn-depleted cell lines (reviewed in Papadimitriou et al., 2010). Indeed, neuroinflammatory gliosis was observed in post-mortem CNS samples from SMA patients (Araki et al., 2003; Garcia-Cabezas et al., 2004; Kuru et al., 2009) with the specific occurrence of reactive astrocytic protrusions, termed glial bundles (Ghatak, 1983; Kuru et al., 2009). Astrocytic activation also occurs in the early stages of disease progression in the spinal cord of severe SMA mice (McGivern et al., 2013; Tarabal et al., 2014). In fact, their cell bodies are enlarged, present an excessive expression of GFAP and develop thick and short processes (McGivern et al., 2013). In addition, the presence of activated microglia has been noted in proximity of motoneurons in the ventral horn area of the spinal cord of severe SMA mice (Ling et al., 2010; Tarabal et al., 2014). Finally, myelinating Schwann cells and nerve capping terminal Schwann cells also display intrinsic defects in SMA mice. A recent analysis of severe SMA mice has demonstrated specific hypomyelination in the proximal intercostal nerve, which innervates pathologically-affected muscles (Hunter et al., 2014). Furthermore, this study reveals that primary Schwann cells isolated from SMA mice have a reduced ability to myelinate healthy axons as well as aberrantly express important myelin proteins such as myelin protein zero (MPZ) and peripheral myelin protein 22 (PMP22). In addition to defects within myelinating Schwann cells, there is a loss of nerve capping terminal Schwann cells at the NMJs of intermediate SMA mice (Murray et al., 2013) that might suggest a defect in migration of Schwann cells to the endplate. While we cannot exclude the fact that Smn depletion may simply reduce the survival of terminal Schwann cells, the former hypothesis is of interest since Smn-depleted astroglioma U897MG cells display a RhoA/ROCK-dependent delay in migration (Caraballo-Miralles et al., 2012). Indeed, these cells express significantly more activated RhoA and downstream effector MLC. Further, the delayed migration in Smn-depleted U897MG cells is reversed by Y-27632, suggesting that the defect is dependent on the RhoA/ROCK pathway. The caveat of this study is that it was performed in a cell line and to date, there is no evidence of glial migration abnormalities in *in vivo* models of SMA or in primary cultures of Smn-depleted astrocytes and Schwann cells. Nevertheless, the studies on U897MG cells and nerve capping terminal Schwann cells (Caraballo-Miralles et al., 2012; Murray et al., 2013) raise the possibility that RhoA/ROCK-dependent glial cell migration is affected in SMA, thus warranting further examination of this aspect in future investigations.

### Potential benefits of ROCK inhibition on glial cells

As discussed above, myelination and migration defects are apparent features of SMA glial cells. Interestingly, migration of Schwann cells along the axon is a necessary step that precedes myelinating events (reviewed in Bradl and Lassmann, 2010). Various studies have demonstrated that an activated RhoA/ROCK pathway is a key inhibitor of Schwann cell migration (Yamauchi et al., 2004; Wang et al., 2013). Thus, systemic administration of Y-27632 or Fasudil to SMA mice may improve Schwann cell migration, subsequently enhancing the myelination potential of Schwann cells and/or adequate nerve capping at the NMJ. Indeed, the migration delay in Smn-depleted U897MG astroglioma cells is corrected following treatment with Y-27632 (Caraballo-Miralles et al., 2012). Alternatively, treating primary dorsal root ganglion (DRG) cultures with Y-27632 promotes neurite outgrowth in a Schwann cell-dependent fashion (Fuentes et al., 2008). Seeing as pre-synaptic NMJ terminals of SMA mice display aberrant remodeling abilities following paralysis (Murray et al., 2013), the combination of improved Schwann cell migration and neuronal outgrowth may explain the specific amelioration of NMJ pathology observed in Y-27632- and Fasudil-treated SMA mice (Bowerman et al., 2010, 2012b). Thus, given the general importance of glial cells for neuronal maintenance, ROCK inhibition, by enhancing both the migration and the myelination of glial cells, could positively affect the function of the surviving motoneurons (Figure 2).

To date, the few references about an astrocytic phenotype in SMA reveal reactive glial bundles in the CNS of SMA patients (Ghatak, 1983; Kuru et al., 2009) as well as activated astrocytes in severe SMA mice during early symptomatic stages (McGivern et al., 2013; Tarabal et al., 2014). Analysis of primary astrocyte cultures shows that RhoA inactivation is necessary and sufficient to induce stellation (Ramakers and Moolenaar, 1998), an actin-dependent morphology representative of *in vivo* astrocytes. Furthermore, treatment of astrocytes with an inhibitor of ROCK (similar to Y-27632) promotes astrocytic stellation (Abe and Misawa, 2003). Administering the Y-27632 or Fasudil compounds to SMA mice may therefore modify the actin cytoskeleton of abnormally activated astrocytes, rendering them less inflammatory. It is important to note however, that the above-mentioned experiments on astrocyte stellation were on wild type and in *in vitro* astrocyte cultures. We thus cannot clearly say how the morphology of activated and *in vivo* astrocytes responds to ROCK inhibition. In addition to morphological changes, Fasudil and Y-27632 also increase glutamate transport in primary astrocyte cultures (Lau et al., 2011). This observation is of interest as defects in glutamate transport have previously been reported in Type 1 SMA patients (Hayashi et al., 2002). Hence, ROCK inhibition could also improve astrocytic uptake of glutamate and as result, reduce glutamate excitoxicity (reviewed in Sattler and Rothstein, 2006).

In microglia, increased activation of the RhoA/ROCK pathway is linked to a reactive phenotype that hinders regeneration following injury. Indeed, inhibition of RhoA signaling in rats following spinal cord injury significantly improves functional recovery (Dergham et al., 2002) as well as decreases the number of RhoA-positive microglia at the site of the lesion (Schwab et al., 2004). Interestingly, activated astrocytes can also recruit reactive microglia by releasing S100 β, a calcium-binding protein (reviewed in Donato et al., 2009), in the CNS environment, which subsequently activates the RhoA/ROCK pathway in microglia and promotes their migration (Bianchi et al., 2011). ROCK inhibition in SMA mice may therefore decrease microgliosis in the spinal cord, thus reducing their contribution to the neurodegenerative process.

While RhoA/ROCK signaling clearly regulates astrocytes, microglia and Schwann cell function (Figure 1), the pathological activation of this pathway has yet to be specifically investigated in SMA glial cells. In addition, the impact of ROCK inhibition on myelination, astrocyte and microglia activation as well as on glial cell migration was not evaluated in Y-27632- and Fasudil-treated SMA mice. These informational gaps thus highlight the need for further exploration of the RhoA/ROCK pathway in glial cell processes in SMA.

## Heart

### RhoA/ROCK, heart physiology and SMA

An important number of reports show that SMA patients present cardiac defects such as arrhythmias or cardiomyopathy, with the most common reported myopathies being atrial and ventricular septum defects and dilated right ventricle (Moller et al., 1990; Burglen et al., 1995; Menke et al., 2008; Rudnik-Schoneborn et al., 2008). Indeed, a study of 63 Type 1 SMA patients revealed that 24% of them presented severe bradycardia (Bach, 2007). Whether these defects are due to impairment of the autonomous nervous system or to cardiomyopathy is presently unknown. Nevertheless, seeing as a significant proportion of SMA patients develop cardiac abnormalities, various groups have evaluated heart pathology in SMA mouse models (Bevan et al., 2010; Heier et al., 2010; Shababi et al., 2010). These independent investigations have found that severe SMA mice display bradyarrhytmia, a lower heart rate, a decreased sympathetic tone, a decreased interventricular septum (IVS) width and an enlargement of the left ventricular septum as well as interstitial fibrosis due to oxidative stress. Specific oxidative stress mediators such as angiotensin II and the nicotinamide adenine dinucleotide phosphate (NADPH) oxidase (Sowers, 2002) are also increased in the hearts of severe SMA mice (Shababi et al., 2010, 2012). Concomitantly, the heart myofibers of SMA mice are disorganized, which could also explain the physiological cardiac defects described herein.

Cardiomyocytes express RhoA and ROCK and this pathway is involved in normal health and function of the heart as well as in cardiovascular diseases (reviewed in Surma et al., 2011) (Figure 1). Indeed, the ROCK signaling cascade, via MYPT1 and MLC2, is involved in stress fiber formation (Amano et al., 1996; Surma et al., 2011). Activation of ROCK also contributes to fibrosis, as *Rock1*^−/−^ mice do not develop ischemia-induced fibrosis (Haudek et al., 2009). Moreover, treating mice with Fasudil inhibits the fibrosis that follows myocardial infarction (Hattori et al., 2004) or constriction of the transverse aorta (Li et al., 2012). The influence of ROCK on fibrosis may be through angiotensin II, as this oxidative stress effector activates the RhoA/ROCK pathway in cardiomyocytes (Aoki et al., 1998). In regards to the regulation of cardiac hypertrophy, a link has been established between ROCK and left ventricular remodeling after myocardial infarction (Hattori et al., 2004) while a study of hypertensive patients with left ventricular hypertrophy reveals that these patients display an increased ROCK activity (Gabrielli et al., 2014). Finally, given that MLC is a substrate of ROCK and that it controls smooth muscle contraction (reviewed by Tsukamoto and Kitakaze, 2013), it would appear evident that the RhoA/ROCK pathway plays a role in cardiac contraction. However, to our knowledge, there is yet to be a study of the impact of ROCK activation and/or inactivation on contractile properties of the heart. One study though has reported increased cardiac contraction following ROCK inhibition in diabetic rats (Lin et al., 2007). Taken together, these known functions of RhoA and ROCK in the heart may underlie some of the cardiac defects observed in SMA patients and mice.

### Potential benefits of ROCK inhibition on cardiomyocytes

The beneficial effect of ROCK inhibition in SMA mice could, at least in part, be due to its effect on cardiac physiology (Figure 2). Previous therapeutic assessments have in fact demonstrated that overall ameliorations in lifespan and typical pathological hallmarks are also accompanied by improved cardiac parameters (Bevan et al., 2010; Shababi et al., 2012).

As discussed above, there is important oxidative stress, fibrosis and hypertrophy within the hearts of SMA mice (Bevan et al., 2010; Heier et al., 2010; Shababi et al., 2010, 2012). Further, seeing as these cardiac components can all be modulated by the RhoA/ROCK pathway (reviewed in Surma et al., 2011), the systemic targeting of ROCK activity in SMA mice could therefore diminish stress fiber formation, fibrosis and size of the left ventricle, thus improving overall cardiac health and function. Indeed, inactivation of ROCK via angiotensin II inhibition prevents stress fiber formation and hypertrophy in a cell line derived from rat heart myoblasts (Kim et al., 2013). Finally, the role of Fasudil as a potent vasodilator (Fukumoto et al., 2005) may also alleviate the fibrosis-dependent vasoconstriction of SMA hearts (reviewed in Wright et al., 2008).

While it is tempting to hypothesize that overexpression of activated RhoA/ROCK during SMA pathogenesis is responsible for the observed cardiac defects, assessment of this pathway in SMA hearts and evaluation of the effects of Y-27632 and Fasudil on cardiac function and pathology have unfortunately not been performed. In addition, since heart abnormalities appear to only occur in severe SMA mouse models and Type 1 patients (Menke et al., 2008), the exact contribution of cardiac dysfunction to SMA disease progression remains unclear and warrants further investigation. Thus, systemic ROCK inhibition may positively alter cardiac function of SMA mice without any direct repercussion on overall survival.

## Pancreas and glucose homeostasis

### RhoA/ROCK, pancreas, glucose metabolism and SMA

Over the years, an accumulating number of studies have reported metabolism defects in SMA patients, which mostly concern glucose metabolism (Lamarca et al., 2013), and fatty acid metabolism (Quarfordt et al., 1970; Dahl and Peters, 1975; Tein et al., 1995; Crawford et al., 1999). More recently, glucose metabolism and pancreatic abnormalities were uncovered in an intermediate SMA mouse model and Type 1 SMA patients such as a dramatic predominance of glucagon-producing α cells at the expense of insulin-producing β cells within pancreatic islets, fasting hyperglycemia, hyperglucagonemia, glucose resistance and increased hepatic insulin sensitivity (Bowerman et al., 2012c). Further, a subset of these pathologies are found in a metabolically-challenged and aging hypomorphic Smn-depleted mouse model that does not display a canonical SMA pathology (Bowerman et al., 2014).

RhoA/ROCK signaling modulates various pathways responsible for pancreatic function and glucose homeostasis (Figure 1). For example, ROCK regulates important parameters of pancreatic β cell function, as treating primary rats β cells with the ROCK inhibitors H-1152 or Y-27632 results in a glucose-independent re-organization of actin cytoskeleton and improved glucose-stimulated insulin secretion (Hammar et al., 2009). Inhibition of the ROCK pathway also increases the proliferation of human pancreatic β cells (Aly et al., 2013) as well as promotes insulin promoter activity and insulin expression in β cell-derived HIT-T15 cells (Nakamura et al., 2006). In addition to a role in pancreatic β cell function, use of a dominant negative ROCK in L6 muscle cells shows that blocking this kinase significantly reduces the insulin-induced glucose transport in myotubes (Furukawa et al., 2005). This study further demonstrated that overexpression of ROCK is sufficient to enhance insulin signaling and response to insulin stimulation in 3T3-L1 adipocytes, L6 myotubes and CHO^*IR/IRS*−1^ cells [CHO cells expressing insulin receptor (IR) and insulin receptor substrate 1 (IRS1)] through phosphorylation of IRS1. All together, these observations point to a key regulatory role for the RhoA/ROCK pathway in specific pancreatic functions as well as in the general maintenance of glucose homeostasis.

### Potential benefits of ROCK inhibition on pancreatic and glucose metabolism function

To date, nothing is known about the effect of SMN depletion on the pancreatic RhoA/ROCK pathway and how it could contribute to SMA pathology. However, beneficial therapeutic strategies evaluated in SMA mice such as muscle-specific insulin-like growth factor 1 (IGF1) administration, neuronal depletion of phosphatase and tensin homolog (PTEN) and systemic trichostatin A administration have previously been shown to also influence glucose metabolism (Di Cola et al., 1997; Ranke, 2005; Stiles et al., 2006; Avila et al., 2007; Sun and Zhou, 2008; Ning et al., 2010; Bosch-Marce et al., 2011). Systemic ROCK inhibition in SMA mice may therefore partially or completely restore secretion of insulin and glucagon by pancreatic cells, glucose tolerance, insulin sensitivity as well as storage of glucose by muscles. Indeed, improvement of the crucial insulin-dependent muscle glucose uptake (reviewed in Huang and Czech, 2007) may explain the significant improvements in skeletal muscle and NMJ pathology observed in Y-27632- and Fasudil-treated SMA mice (Bowerman et al., 2010, 2012b). Alternatively, the positive effect of ROCK inhibition could be directly achieved through pancreatic modulation as inactivation of the ROCK pathway enhances β cell proliferation, insulin expression and glucose-stimulated insulin secretion (Nakamura et al., 2006; Hammar et al., 2009; Aly et al., 2013). However, these results were obtained in β cell cultures and whether systemic inhibition of ROCK would have the same effects *in vivo* still needs to be evaluated. In the same manner, the observation that ROCK enhances insulin signaling and glucose storage in muscle cell lines should be verified in animal models. Lastly, it has been reported that β cell proliferation is modulated by an interaction between CREB, a transcription factor that mediates hepatic glucagon signaling (reviewed in Quesada et al., 2008; Altarejos and Montminy, 2011), and the RhoA/ROCK pathway (Aly et al., 2013). Combined with the recent observation that hepatic p-CREB signaling is increased in various Smn-depleted mouse models (Bowerman et al., 2014), these findings strongly support the assessment of the RhoA/ROCK pathway in all SMA tissues (pancreas, liver, muscle) that coordinate glucose homeostasis.

## Conclusion and perspectives

While SMA is undeniably primarily a motoneuron disease, a growing number of studies have reported defects in other cell types and organs, whether they be within the CNS such as glial cells, or outside, like the pancreas, heart or skeletal muscle (reviewed in Hamilton and Gillingwater, 2013). The development of systemic therapeutic approaches such as ROCK inhibition has further emphasized the need to further understand the contribution of multiple cell types in this pathology. In the present review, we have described the known roles of the RhoA/ROCK pathway in several cells and organs that have previously been reported to be affected in SMA cellular and mouse models as well as in patients (Figure 1). We have further hypothesized how systemic ROCK inhibition could influence each particular cell and/or tissue eventually impacting disease progression in SMA mice (Figure 2).

In neuronal cells, the primary cell type affected in SMA, inhibiting ROCK could render neurons more plastic, thus permitting a better maintenance of functional NMJs. Skeletal muscle for its part, is also an important tissue proven to play both intrinsic and extrinsic roles in SMA pathology (reviewed in Boyer et al., 2013). ROCK inhibition in skeletal muscle could help restore normal myogenesis and developmental programs. Astrocytes, microglia and Schwann cells are essential glial cells required for the proper maintenance of CNS homeostasis (reviewed in Fields and Stevens-Graham, 2002; Allen and Barres, 2009) and recent studies suggest that they may be aberrantly regulated in SMA. Indeed, SMA glial cells show impaired myelinating capacity, decreased migration, increased activation and reduced number at the NMJ (Caraballo-Miralles et al., 2012; McGivern et al., 2013; Murray et al., 2013; Hunter et al., 2014; Tarabal et al., 2014), all of which could be ameliorated following systemic ROCK inhibition. SMA patients and mouse models also display important cardiac defects such as oxidative stress, fibrosis and hypertrophy (Moller et al., 1990; Burglen et al., 1995; Bach, 2007; Menke et al., 2008; Rudnik-Schoneborn et al., 2008; Bevan et al., 2010; Heier et al., 2010; Shababi et al., 2010), pathological events controlled by the RhoA/ROCK pathway (reviewed in Surma et al., 2011). ROCK inhibition could therefore have a positive effect on overall cardiac physiology and function in SMA mice. Finally, SMA patients and mice show defects in glucose and fatty acid metabolism as well as in pancreatic islet development (Quarfordt et al., 1970; Dahl and Peters, 1975; Tein et al., 1995; Crawford et al., 1999; Bowerman et al., 2012c, 2014; Lamarca et al., 2013). Given that the RhoA/ROCK pathway enhances β cell proliferation, insulin expression, glucose-stimulated insulin secretion and glucose uptake in muscle cells (Furukawa et al., 2005; Nakamura et al., 2006; Hammar et al., 2009; Aly et al., 2013), treatment of SMA mice with ROCK inhibitors may modulate key glucose homeostasis pathways.

It thus appears that it has become insufficient to limit our evaluation of therapeutic approaches for SMA to neuronal function and pathology. While SMA remains a canonical neurodegenerative disease, it is becoming more and more evident that a convergence of multiple pathways, including RhoA/ROCK, in various cells and tissues within or outside the CNS, influence SMA pathogenesis and have to be taken in account when assessing the beneficial effects of systemically-delivered pharmacological compounds.

Of both Y-27632 and Fasudil, only Fasudil has been approved for evaluation in clinical trials for disorders such as Raynaud's Phenomenon, atherosclerosis and amyotrophic lateral sclerosis (ALS), a motoneuron pathology closely related to SMA (reviewed in Achsel et al., 2013). This is of further relevance due to the recent identification of mutations within the *Profilin 1* gene in ALS patients (Wu et al., 2012), highlighting profilin, a downstream effector of ROCK, as a common pathological player in both ALS and SMA. However, to our knowledge, there are currently no planned clinical trials of Fasudil for SMA patients. One of the reasons may be the potential toxicity of this compound in younger animals (Momma et al., 2009; Bowerman et al., 2012b). For the Y-27632 study in SMA mice, a low (10 mg/kg) and high (30 mg/kg) daily dose was based on previous dosing regimens validated in adult mice following cardiac ischemia/reperfusion injury (Bao et al., 2004). As for Fasudil in SMA mice, previously validated low (30 mg/kg) and high (100 mg/kg) (Fukui et al., 2008) daily doses were also initially used (Bowerman et al., 2012b). However, the lower dose proved to have no effect, most likely due to its short half-life and rapid metabolization (Satoh et al., 2001), while the higher dose was lethal in neonatal pups, probably because of Fasudil's role as a potent vasodilator (Fukumoto et al., 2005). In an attempt to both maximize the activity of Fasudil and reduce its toxicity, an escalating dosing regimen (30 mg/kg twice daily from post-natal day (P) 3 to P6; 50 mg/kg twice daily from P7 to P13; 75 mg/kg twice daily from P14 to P21) was administered to SMA mice (Bowerman et al., 2012b). This however, also led to a non-negligible lethality. Thus, an intermediate dose of 30 mg/kg twice daily was finally evaluated, which resulted in the beneficial effects described herein. Seeing as SMA mostly affects children under the age of two (Crawford and Pardo, 1996), the reported toxicity of Fasudil (Bowerman et al., 2012b; Momma et al., 2009) will have to be seriously taken into account when establishing a clinical trial of ROCK inhibitors for SMA patients. The recently started clinical trial of Fasudil for ALS patients is nevertheless at step forward for the therapeutic assessment of ROCK inhibitors in neurodegenerative diseases and will, without a doubt, help pave the path for similar endeavors in SMA.

### Conflict of interest statement

The authors declare that the research was conducted in the absence of any commercial or financial relationships that could be construed as a potential conflict of interest.
